# Metabolic Profiling of Early and Late Recurrent Pancreatic Ductal Adenocarcinoma Using Patient-Derived Organoid Cultures

**DOI:** 10.3390/cancers12061440

**Published:** 2020-06-01

**Authors:** Lukas M. Braun, Simon Lagies, Rhena F. U. Klar, Saskia Hussung, Ralph M. Fritsch, Bernd Kammerer, Uwe A. Wittel

**Affiliations:** 1 Center for Biological Systems Analysis ZBSA, Albert-Ludwigs-University Freiburg, 79104 Freiburg, Germany; lukas.braun@uniklinik-freiburg.de (L.M.B.); simon.lagies@zbsa.uni-freiburg.de (S.L.); 2 Department of General and Visceral Surgery, University of Freiburg, Medical Center Faculty of Medicine, 79106 Freiburg, Germany; 3 Institute of Biology II, Albert-Ludwigs-University Freiburg, 79104 Freiburg, Germany; 4 Spemann Graduate School of Biology and Medicine, Albert-Ludwigs-University Freiburg, 79104 Freiburg, Germany; 5 Department of Medicine I (Hematology, Oncology and Stem Cell Transplantation), University of Freiburg Medical Center, 79104 Freiburg, Germany; rhena.klar@uniklinik-freiburg.de (R.F.U.K.); saskia.hussung@uniklinik-freiburg.de (S.H.); ralph.fritsch@usz.ch (R.M.F.); 6 SFB/Collaborative Research Center 850 (CRC 850)—Control of Cell Motility in Morphogenesis, Cancer Invasion and Metastasis, University of Freiburg, 79104 Freiburg, Germany; 7 German Cancer Consortium, 79104 Freiburg, Germany; 8 Comprehensive Cancer Center Freiburg, 79104 Freiburg, Germany; 9 Department of Medical Oncology and Hematology, University Hospital of Zurich, 8091 Zurich, Switzerland; 10 BIOSS Centre for Biological Signalling Studies, University of Freiburg, 79104 Freiburg, Germany

**Keywords:** pancreatic ductal adenocarcinoma, PDAC, recurrence, relapse, metabolomics, GC/MS, patient derived organoids, PDO culture, anaplerotic TCA-cycle, glutamate

## Abstract

Pancreatic ductal adenocarcinoma (PDAC) is associated with high mortality and will become the second most common cause of cancer-associated mortality by 2030. The poor prognosis arises from a lack of sensitive biomarkers, limited therapeutic options, and the astonishingly high recurrence rate after surgery of 60–80%. The factors driving this recurrence, however, remain enigmatic. Therefore, we generated patient-derived organoids (PDOs) from early- and late-recurrent PDAC patients. Cellular identity of PDOs was confirmed by qPCR, ddPCR, and IHC analyses. This is the first study investigating the metabolism in PDOs of different, clinically significant PDAC entities by untargeted GC/MS profiling. Partial least square discriminant analysis unveiled global alterations between the two sample groups. We identified nine metabolites to be increased in early recurrent PDOs in comparison to late recurrent PDOs. More than four-times increased were fumarate, malate, glutamate, aspartate, and glutamine. Hence, α-keto acids were elevated in PDO-conditioned medium derived from early recurrent patients. We therefore speculate that an increased anaplerotic metabolism fuels the Krebs-cycle and a corresponding higher accessibility to energy fastens the recurrence in PDAC patients. Therein, a therapeutic intervention could delay PDAC recurrence and prolong survival of affected patients or could serve as biomarker to predict recurrence in the future.

## 1. Introduction

Pancreatic ductal adenocarcinoma (PDAC) is the most common histological subtype of pancreatic cancer and a highly aggressive and fast-growing type of cancer [1,2,3]. In about 90% to 95 % of all pancreatic tumors, the cell-of-origin can be found in the pancreas ductal epithelium [1]. PDAC is associated with a very poor prognosis [4], is one of the major causes for cancer-related death in the western world [5] and is predicted to become the second-most cause of cancer-associated mortality [6] by 2030 [7]. The 5-year survival rate of PDAC patients has changed only little during recent years [5] and is now around 10% for all stages [8,9]. Important risk factors for PDAC include genetic predisposition including germline mutations in *BRCA2* [10], *CDKN2A* [11], and *CFTR* [12] as well as smoking, diabetes, obesity and chronic pancreatitis [4,13]. The very low survival rates associated with PDAC are due to several factors, of which the most important are the very rapid progression, early microdissemination [14], and the late onset of any specific symptoms [15]. The majority of PDACs are diagnosed at an advanced, locally irresectable or metastatic stage when only palliative therapies can be offered [16]. Biologically, pancreatic cancers are locally highly infiltrative tumors with vascular and perineural invasion being observed frequently. Moreover, locoregional lymphatic and/or distant metastases are found at first diagnoses in the majority of cases [17]. The molecular pathology of PDAC is extremely challenging with a 92–95% prevalence in non-druggable activating *KRAS* mutation next to *TP53* and/or *CDKN2A* alterations driving cancer survival, growth, resistance to therapeutic options and metastasis [18]. These factors further contribute to a rapid progression of the disease [19] and support anabolic metabolic pathways [15]. About 80% of all patients suffering from pancreatic cancer have unresectable carcinoma at diagnosis, decreasing their estimated overall survival to only a few months [20]. The therapeutic options for these patients are limited to combination chemotherapy, which is associated with significant toxicity [15]. Many patients who undergo curative resection experience disease recurrence within one year after surgery [21,22,23,24]. 

For patients initially presenting with surgically resectable PDAC (approximately 20%) [25], disease recurrence after initial curative surgery is the major challenge and impediment to long-term survival. Among all patients undergoing resection, cancer recurrence develops in up to 80% within time frames of clinical trials and are probably even higher in real-life populations [26,27,28]. Many studies found that the early post-operative recurrence is due to local and distant metastases [29]. Patients who developed distant metastases early after surgery have only a limited median survival time of a few months [30,31]. Studies trying to identify risk factors for recurrence after PDAC resection found tumor size, carbohydrate antigen 19-9 (CA19-9), perineural invasion, and lymph node metastases to correlate with disease recurrence [32,33,34]. However, it is likely that microscopic metastases have formed in the majority of patients prior to surgery or radiation therapy [15]. However, the mechanisms determining early or late PDAC recurrence after surgery have not yet been extensively studied [29,30,31] and better predictive biomarkers are required for more successful clinical stratification of patients.

During the last decades, many attempts in pancreatic cancer research focused on understanding molecular mechanisms within the tumor cells, the development of pancreatic cancer, and finding new therapeutic options to overcome treatment resistance [35]. Moreover, different studies tried to find novel biomarkers for the early detection of pancreatic cancer [36,37,38]. However, the use of the limited number of established pancreatic cancer cell lines in these studies was associated with several limitations. First, many cell lines were established from metastatic sites and do underrepresent characteristics found in primary tumors or pancreatic intraepithelial neoplasia (PanIN) lesions [39], and second, established cell lines grow extremely fast which could easily lead to the selection of different clones and a genetic drift as reported previously [40]. Application of 3D-grown conventional cell lines can in general overcome some issues of abnormal proliferation behavior observed in 2D-cultures in comparison to in situ situations [41]. Other research systems include patient-derived xenografts (PDX). However, these systems require large amounts of tissue [42] and need several months to be established [43]. Genetically engineered mouse models of PDAC could give good insights into the disease, but the systems are time-consuming, need a lot of space, and require high costs [44]. In order to overcome at least some of the limitations, 3D patient-derived pancreatic ductal organoids (PDO) can be cultured directly from primary PDAC tissue, pancreatitis tissue, or even healthy pancreas [45]. Organoids are a cellular 3D system with self-renewal and self-organization capacity which maintains an appearance and functionality comparable to their original tissue [39]. Moreover, the systems were found to be genetically stable throughout many passages [46,47]. Human PDAC organoids could generate PanIN lesions and develop into invasive PDAC after transplantation into mice [45]. With PDO cultures, PDAC of a variety of patients can be modeled in vitro and experimental findings can be correlated to the clinical course of the donating patients [45]. The organoid culture system allows the expansion of primary tissue in an in vitro system which is much closer to the actual patient than established cell lines are. Moreover, these systems allow the growth of 3D structures from healthy and disease tissue making the direct comparison between both states more convenient [48]. One of the major current limitations with organoid cultures from patient tissue is the limitation in cell expansion and the heterogenic growth [48]. The cultures need relatively long to grow and to deliver sufficient cell mass, compared to 2D cultures of established cell lines. Moreover, the organoid cultures depend on many supplemental factors to grow in vitro [39]. 

In this study, we generated different PDOs from early and late recurrent PDAC patients. After the evaluation of PDOs in regard to tissue-specificity, differences in primary metabolism between early and late recurrence should be unveiled by untargeted GC/MS profiling. We found that PDOs derived from early recurrent PDAC patients had higher levels of tricarboxylic acid intermediates and some anaplerotic amino acids, suggesting a higher capacity to generate energy. Thus, early recurrence might be the consequence of higher energy levels in these malignancies, suggesting a possible target to delay recurrence in future PDAC therapy or potential biomarkers to predict recurrence.

## 2. Material and Methods

### 2.1. Preparation of Human Pancreas Organoid Feeding Medium

Feeding medium for human pancreas organoid cultures (PDO) was prepared freshly at least every two weeks. The organoid splitting medium was prepared by supplementing Advanced DMEM/F-12 medium with 10 mM HEPES and 1× GlutaMAX™. The ROCK inhibitor Y-27632 (10.5 µM) was added to the feeding medium directly before use. For organoid feeding medium, following ingredients were added to the splitting medium (final concentrations are given): 100 µg/mL primocin, 500 nM A38-01, 50 ng/mL mEGF, 100 ng/mL hFGF10, 10 nM gastrin I, 1.25 mM N-acetylcysteine, 10 mM nicotinamide, 1× B27 supplement, 1× R-Spondin I-conditioned medium, 1× Wnt3a-conditioned medium and 10.5 µM Y-27632.

### 2.2. Cultivation and Passaging of Human Pancreas Organoid Cultures

PDAC organoids were established [49] and cultured as described previously [45]. Organoids were grown in 25 µL Matrigel Matrix Basement Membrane domes in 24-well plates. Cultures were passaged once a week and split 1:2 if well-growing. Feeding medium was replaced twice a week. For organoid passaging, matrigel domes were transferred into 10 mL cold splitting medium and kept on ice until centrifugation. All centrifugation steps were performed at 1200 rpm and 4 °C for 5 min. Organoids were centrifuged, splitting medium was discarded, cells were resuspended in 1 mL TrypLE Express (RT), and incubated at 35 °C and 180 rpm for 15 min. Following, 9 mL of cold splitting medium were added into each tube and cells were centrifuged again. Organoid cell pellets were subsequently resuspended in cold Matrigel and immediately seeded into 24-well plates with 25 µL Matrigel for each dome. Organoids were incubated at 37 °C for 15 min before 500 µL feeding medium were carefully added into each well. Organoids were cultured at 37 °C and 5% CO_2_ in a humidified atmosphere.

### 2.3. Isolation of Total RNA from Organoid Cultures

For isolation of RNA from organoid cultures, matrigel domes were resuspended in 1 mL ice-cold 1× PBS. The organoids were centrifuged at 5500 rpm and 4 °C for 6 min. The organoid pellets were resuspended in 500 mL Cell Recovery Solution and incubated on ice for 60 min. Following, the organoids were centrifuged (same settings as before) and washed once with 500 µL ice-cold 1× PBS. The supernatant was discarded and the organoid pellets were immediately frozen at −80 °C. RNA was isolated using the RNeasy^®^ Plus Mini Kit (Qiagen, Hilden, Germany) and QIAshredder (Qiagen) according to manufacturer’s instructions. Briefly, organoid pellets were resuspended in 350 µL Buffer RLT Plus, loaded on a QIAshredder column and centrifuged at 13,000 rpm for 2 min. The homogenate was transferred to a gDNA Eliminator spin column in a new collection tube and centrifuged for 30 s at 10,000 rpm. A total volume of 525 µL 100% ethanol was added to the flow-through and shortly vortexed. 700 µL of the mixture were loaded onto an RNeasy Mini spin column and centrifuged at 10,000 rpm for 30 s. The flow-through was discarded and the step was repeated until the whole sample passed the membrane. Following, 500 µL Buffer RPE was added to the spin column and centrifuged at 10,000 rpm for 30 s. The flow-through was discarded and the step was repeated once. The mini spin column was centrifuged at 13,000 rpm for 2 min to dry the membrane. The column was placed in a new collection tube, 35 µL RNase-free water were added onto the membrane and columns were incubated at RT for 3 min. Following, samples were centrifuged at 13,000 rpm for 2 min to elute total RNA. 

### 2.4. Synthesis of cDNA from RNA Templates

The cDNA from RNA templates was synthesized using the RevertAid First Strand cDNA Synthesis kit (Thermo Scientific, Waltham, MA, USA). Briefly, 1 µg total RNA was incubated with 0.5 µL OligodT and 0.5 µg Random Hexamer primer for 5 min at 70 °C in a total volume of 12 µL. Following, 4 µL Reaction Buffer (5×), 2 µL dNTP mix and 1 µL RNase inhibitor were added and the mix was incubated for 5 min at 37 °C. After addition of 1 µL RevertAid transcriptase, the reaction was run for 10 min at 25 °C, 60 min at 42 °C and 10 min at 70 °C. The cDNA was diluted to 3 ng/µL for qPCR analysis. Additionally, total RNA was diluted to 3 ng/µL for noRT-control.

### 2.5. Design of Primer Sequences for qPCR

Primers were designed for use with SYBR Green in qPCR assays. The mRNA sequences of the respective genes were downloaded from NCBI/gene website and primers were designed using the primer-BLAST tool (NCBI, Bethesda, MD, USA). All primers were designed with a GC content from 40% to 60%, a melting temperature between 58 °C and 60 °C with an optimum at 60 °C and a primer length between 18 bp and 22 bp with an optimum at 20 bp. Sequences are shown in Table S1.

### 2.6. Quantitative PCR (qPCR) Analysis

For analysis of relative gene expression of epithelial and fibroblast markers in PDOs and cell lines, primers were diluted to 7.5 µM in H_2_O. A total amount of 9 ng cDNA or noRT-control was loaded together with 0.5 µL fwd-primer, 0.5 µL rev-primer and 5 µL Power SYBR Green PCR Master Mix (Thermo Scientific) in a final volume of 10 µL in triplicates in 384-well qPCR plates. Negative controls lacking cDNA were included in the assay. The qPCR was run with the LightCycler 480 Instrument II (Roche, Basel, Switzerland) using the following program: 10 min at 95 °C, followed by 40 cycles denature for 15 s at 95 °C and annealing for 60 s at 60 °C. The relative expression of target genes was normalized to β-actin expression and calculated as fold-change over all samples using the 2^−∆∆Ct^ method.

### 2.7. Isolation of DNA from Organoid Cultures

For DNA isolation from the organoid cultures, matrigel domes were each resuspended with 1 mL ice-cold 1× PBS and transferred to an Eppendorf tube. The organoids were then centrifuged at 5300 rpm and 4 °C for 5 min. The supernatant was discarded and the organoid pellet was resuspended in 500 µL Cell Recovery Solution and incubated on ice for one hour. Organoids were then centrifuged again at 5300 rpm and 4 °C for 5 min and the supernatant was removed. The pellet was washed with 500 µL ice-cold 1× PBS and centrifuged for 10 min at 5300 rpm and 4 °C. The supernatant was discarded and the organoid pellet was frozen at −80 °C until DNA isolation. DNA was isolated with the QIAamp DNA Micro Kit (Qiagen) using the protocol “Isolation of Genomic DNA from Tissues” according to the manufacturer’s instructions. DNA was frozen at −20 °C until subsequent ddPCR analyses. 

### 2.8. Droplet Digital PCR (ddPCR)

Locked nucleic acid (LNA) probes and corresponding primer pairs for *KRAS* mutations were designed using Beacon Designer v.8.20 software (Premier Biosoft, Palo Alto, CA, USA). Primers and probes were manufactured by Integrated DNA Technologies (IDT, Inc., Coralville, IA, USA). Detailed information on primer and probe design as well as the corresponding sequences have been published previously [50]. Primers, probes, 2 µL/well template DNA, and nuclease-free water (Ambion) were added to ddPCR Supermix for Probes (Bio-Rad, cat. no #186-3024). Reaction mix was set up as recommended and droplets were generated using a QX100/200TM Droplet Generator (Bio-Rad, cat. no. #1863002) following manufacturer’s instructions. Each sample was assayed in quadruplicates. Following Droplet Generation, droplets were transferred into a 96-well PCR plate (Bio-Rad, cat. no. #12001925). PCR was run on a C1000 TouchTM Thermal Cycler (Bio-Rad, cat. no. #1851197), and samples were subsequently analyzed on a QX100/200TM Droplet Reader (Bio-Rad, cat. no. 1863003) using QuantaSoft v1.7.4.0917 (Bio-Rad, cat.no. #1864011). PCR protocols for the corresponding *KRAS* assays, the respective assay controls, and data analysis were performed as described previously [50].

### 2.9. Embedding Organoids for Immunohistochemistry (IHC) Analysis

For IHC analysis, organoid domes were fixed in 500 µL 4% paraformaldehyde (PFA) solution at RT for 15 min after removal of feeding medium. PFA was removed and domes were embedded in 1 mL of 2% agarose (*w/v*; in water) solution. After polymerization, the embedded domes were stored in 50% ethanol (*v/v*) at 4 °C until further processing. The domes were embedded in paraffin, cut into 3 µm sections and placed on coverslips.

### 2.10. Immunohistochemistry Staining

For IHC staining, sections were incubated overnight at 37 °C, deparaffinized and boiled in a pressure cooker in 10 mM citrate buffer (pH 6.0, supplemented with 0.05% Tween20) for 15 min. Endogenous peroxidase activity was quenched with peroxidase-blocking-solution for 30 min, followed by 3 × 5 min wash in TBS-T (pH 7.6, 0.05% Tween20). The background was blocked with 1% BSA/TBS for 30 min. Primary antibodies were diluted in 1% BSA/TBS (αSMA 1:400, CK19 1:400, PDX1 1:1000, Vimentin 1:400) and slides were incubated with the respective antibodies overnight at 4 °C. Slides were washed 3 × 5 min in TBS-T and incubated with EnVision+/HRP-labeled polymer anti-rabbit or anti-mouse for 60 min at RT. Slides were washed again 3 × 5 min in TBS-T. Stainings were visualized by incubation with the Liquid DAB+ Substrate Chromogen System (DAKO, 1:51) for 2 min, followed by washing in water. Counter stain was done with Mayer’s hematoxylin (1:5, *v/v*, in water) for 5 min, followed by incubation in water (37 °C) for 10 min. The coverslips were mounted using the Rotihistokitt II (Roth, Karlsruhe, Germany). Details of the antibodies used in this study are depicted in Table S2.

### 2.11. Organoid Harvest and Metabolite Extraction

Organoids were passaged as described previously and cultured for eight days until matrigel domes were confluent for harvesting. The plates were placed on ice, the medium was removed and the matrigel domes were disrupted in 1 mL ice-cold 0.9% NaCl solution by thoroughly pipetting up and down and transferred into 15 mL canonical tubes. Organoids from three domes were pooled for one analysis sample and washed thrice with 10 mL ice-cold 0.9% NaCl solution to remove the matrigel from the cells. The metabolism was quenched by the addition of 1.5 mL ice-cold extraction buffer (MeOH/H2O 9:1 (*v/v*), 1 µg/mL ribitol, 1 µg/mL phenyl-β-d-glucopyranoside) and the suspension was transferred into 2 mL screw-cap-tubes which had been pre-filled with 300 mg glass beads. The cells were snap-frozen in liquid nitrogen and stored at −80 °C until further processing. The cell samples were applied to a Precellys Evolution tissue homogenizer for cell lysis with the following settings: 3 × 15 s at 6500 rpm with a 10 s break between the homogenization steps. The samples were subsequently centrifuged at 20,000 rcf and 4 °C for 10 min to separate cell debris. 1200 µL of the supernatant were dried under vacuum and analyzed by GC/MS.

### 2.12. Metabolite Extraction from PDO Conditioned Medium

Proteins were precipitated and metabolites extracted by cold acetonitrile:methanol as described in Lagies et al. [51]. Vacuum dried metabolite pellets were subjected to GC/MS analysis.

### 2.13. GC/MS Based Metabolic Profiling

Dried metabolite pellets were subjected to untargeted gas chromatography coupled to mass spectrometry (GC/MS) based profiling as described previously [51]. In summary, metabolites were derivatized by methoxyamine hydrochloride in pyridine followed by silylation with *N*-trimethylsilyl-*N*-methyl trifluoroacetamide. Samples were splitlessly injected onto an HP-5MS column (60 m × 0.25 mm × 0.25 µm) in randomized order with regular quality control samples in between. Metabolites were analyzed by electron ionization and annotated according to retention index and mass spectral similarity to different libraries, including an in-house database. Features were aligned by SpectConnect and normalized to internal standard and peak-sum. Statistical analysis was conducted with MetaboAnalyst 4.0. 

### 2.14. Statistical Analysis

For others than untargeted metabolomics profiling data, statistics were performed using the GraphPad prism software. Normally distributed data were compared using an unpaired *t*-test. Data are displayed as mean ± standard error of the mean. A *p*-value < 0.05 was considered as statistically significant. Significances were shown with symbols (*: *p* <0.05, **: *p* < 0.01, ***: *p* < 0.001).

### 2.15. Data Avialbility

Results and statistical results of metabolic profiling are provided in supplementary data files Tables S3 and S4 for endometabolites and exometabolites, respectively. Other data will be available upon reasonable request.

### 2.16. Compliance with Ethical Standards

Informed consent was obtained for the development and usage of 3D Organoid cultures from human pancreatic cancer tissue and approved by the local Ethics Committee of the Albert-Ludwigs-University Freiburg, Freiburg, Germany (126/17; 28 March 2017).

## 3. Results

### 3.1. Patient and Clinical Data of Organoid Lines Used in This Study

Patient-specific data of the patient-derived organoid lines used in this study are summarized in Table 1. The table contains information about patient sex and year of birth, additional to clinical information about the tumor grading, the KRAS mutation, and the recurrence state of the tumor. “Early recurrence” summarizes tumor recurrence within the first six months after curative resection, whereas “late recurrence” indicates disease recurrence more than six months after surgery. 

### 3.2. Analysis of Pancreas and Fibroblast Lineage Marker Expression

**Figure 1:**
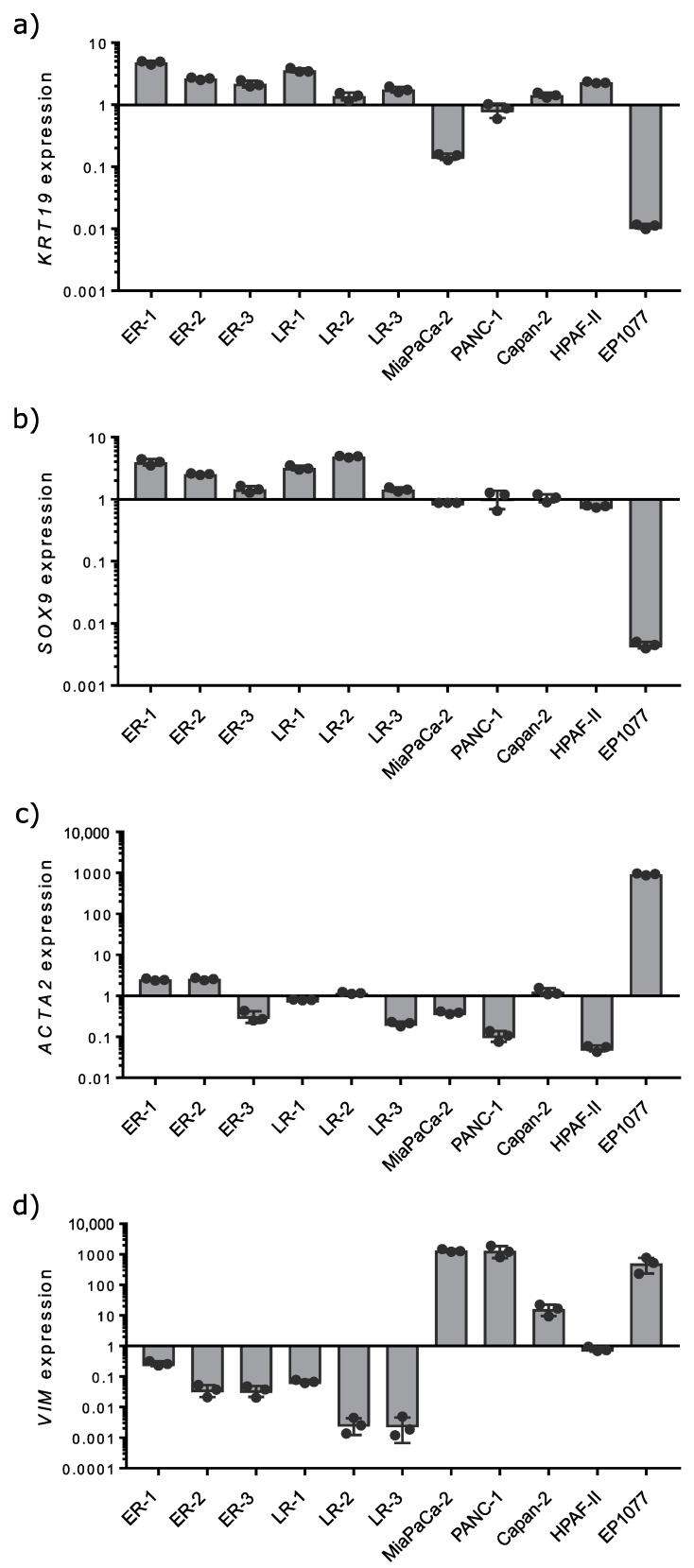
Gene expression analysis of epithelial, ductal and mesenchymal markers in PDOs, PDAC cell lines and PSCs. The expression of cytokeratin 19 (*KRT19*; **a**), *SOX9* (**b**), alpha smooth muscle actin (*ACTA2*; **c**) and vimentin (*VIM*; **d**) was analyzed by qPCR and normalized to β-actin expression. Gene expression was analyzed in early and late recurrent PDOs as well as in a pancreatic stellate cell line (EP1077) and the established PDAC cell lines Capan-2, HPAF-II, MiaPaCa-2 and PANC-1. Results are depicted in a logarithmic scale as fold change over all samples. Bar charts display mean ± standard error of the mean (*n* = 3).

In order to control for the presence of pancreatic ductal cells versus fibroblasts in organoid cultures, the expression of lineage markers for both cell types was analyzed by qPCR (Figure 1). Gene expression was analyzed in PDO cultures, in myofibroblast-like pancreatic stellate cells (PSC, EP1077) and the established PDAC cell lines Capan-2, HPAF-II, MiaPaCa-2, and PANC-1 (PDAC). Expression is shown as fold-change over all samples. Most of the organoid lines expressed the ductal lineage markers cytokeratin 19 (*KRT19*) and SRY-Box transcription factor 9 (*Sox9*). Expression in PDAC cell lines was lower. RNA derived from PSC was used as a negative control for these markers. The results indicated a high content of ductal adenocarcinoma cells in the organoid cultures. Additionally, the expression of alpha smooth muscle actin (*ACTA2*) and vimentin (*VIM*) was analyzed as markers for mesenchymal cells and fibroblasts in the cultures. *ACTA2*, a marker for activated pancreatic stellate cells and cancer-associated fibroblasts, was detected in all organoid lines at very low levels. A high expression was found in PSC, serving as a positive control for fibroblast markers. Additionally, vimentin expression was low in all organoid lines, except for the chronic pancreatitis and the pseudocyst samples. Vimentin is a typical marker for mesenchymal-like cells. The high expression of vimentin in PDAC cell lines confirmed their mesenchymal type described previously [52].

Next, we assessed the mutation allele frequencies (MAFs) of the *KRAS*-mutations by ddPCR. All PDO harbored high fractions of the corresponding *KRAS*-mutation (Figure S1). Hence, these fractions were starkly elevated in comparison to the primary biopsies, indicating a very pure tumor population.

**Figure 2:**
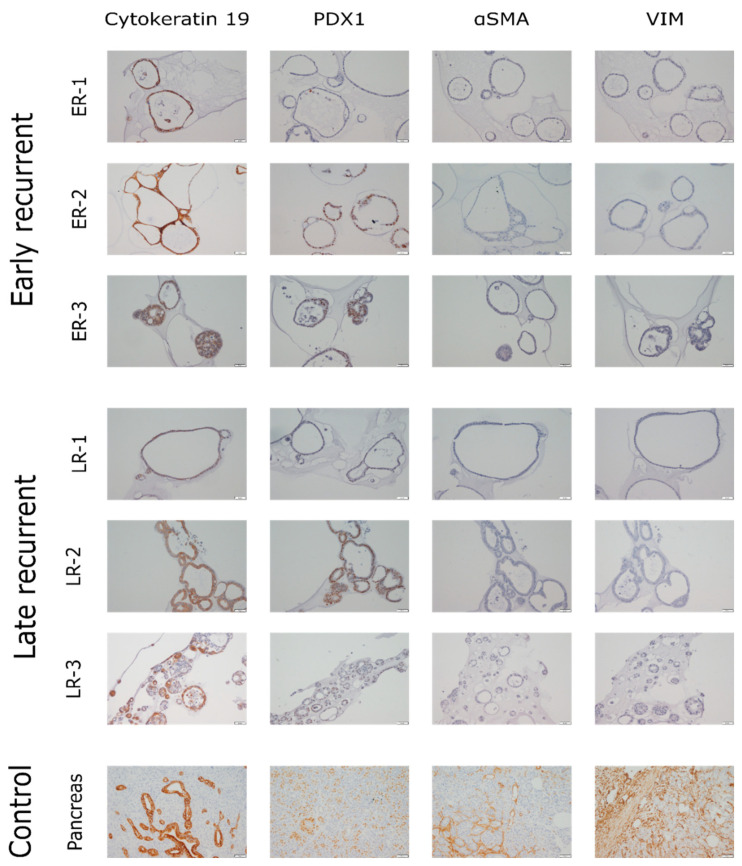
Immunohistochemistry staining of PDOs. Paraffin-embedded organoids were cut into 3 µm sections and stained with antibodies against CK19 (first column) or PDX1 (second column) as markers for epithelial and ductal cells as well as antibodies against αSMA (third column) or vimentin (fourth column) as markers for fibroblasts and mesenchymal cells. Brown color represents a positive signal. Pancreatic tissue served as control. Each panel depicts approximately 500 × 675 µm.

Besides gene expression analysis, lineage markers were also analyzed by immunohistochemistry staining. PDO sections were stained with antibodies against cytokeratin 19 (CK19) and pancreatic and duodenal homeobox 1 (PDX1) as epithelial and pancreas duct lineage markers (Figure 2, left panels), as well as with antibodies against alpha smooth muscle actin (αSMA) and vimentin as fibroblast and mesenchymal lineage markers (Figure 2, right panels). The images are grouped according to the clinical group used for analysis. Pancreas tissue served as control. CK19 was found at high levels in all samples. Levels of PDX1 were much more variable with low and high expressing lines in all groups. ER-1 (early recurrent) and LR-1 (late recurrent) displayed lower levels of PDX1 compared to all other lines. Staining against αSMA and vimentin revealed a low level or even negative staining in all PDOs. The findings are in line with qPCR results. In conclusion, all organoid lines contained high amounts of epithelial and ductal cells, confirming them as a suitable system to study pancreatic ductal adenocarcinomas in vitro. However, also fibroblast markers were found, representing either some fibroblasts in PDO cultures or an EMT phenotype, as reported for PDAC cell lines [52].

### 3.3. Metabolic Analysis of PDOs Derived from Early and Late Recurrent PDAC Patients

**Figure 3:**
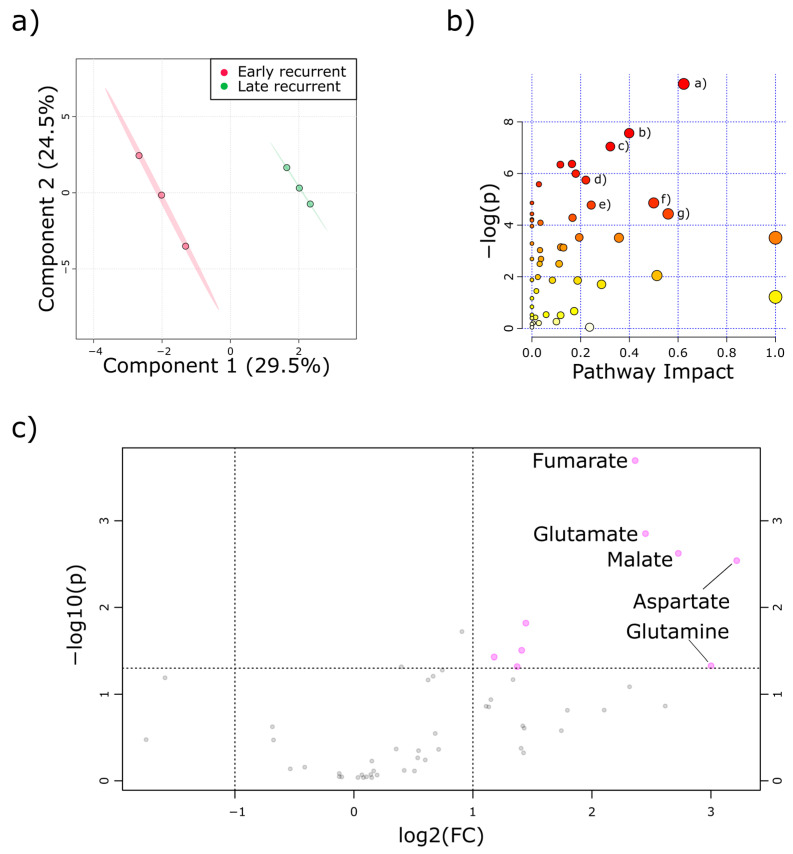
Metabolic profiling revealed tricarboxylic acid cycle (TCA-cycle) intermediates and anaplerotic amino acids were increased in early recurrent PDAC. (**a**) Partial least square discriminant analysis of PDOs derived from early (red) and late (green) recurrent PDAC patient unveiled global metabolic alterations. (**b**) Global pathway analysis revealed different pathways affected in PDOs. Identity of the pathways matching the cut-off values (−log(*p*) > 4 and pathway impact > 0.2) are depicted in table 2. (**c**) Volcano plot analysis of identified metabolites. Early recurrent to late recurrent fold changes are displayed as log2 values. Only top five altered metabolites are labeled. Each data point represents at least three technical replicates of the different PDOs.

Following validation of the organoid cultures, untargeted metabolomics analysis was conducted in early passage PDOs. For that, each PDO culture was measured in at least three technical replicates. As early and late recurrent PDAC patients could clearly be assigned to different clinical entities, we conducted a partial least square discriminant analysis (PLS-DA). Indeed, PLS-DA revealed that there were significant global alterations within the two metabolite pools, as shown by separated confidence intervals (see Figure 3a).

Next, we analyzed which metabolic pathways were differently regulated in early and late recurrent PDOs. Indeed, several pathways were affected when comparing early and late recurrent PDAC patients (see Figure 3b). Table 2 summarizes the pathway entities from Figure 3b with corresponding pathway impact and statistical evaluation. More than half of the most affected pathways regulated differently in early and late recurrent PDOs were the TCA-cycle itself or anaplerotic pathways, closely connected to the TCA-cycle. 

To further elucidate which metabolites contributed most to this separation, we conducted a volcano-plot analysis (Figure 3c). Fumarate and malate were the most increased TCA-cycle intermediates in PDOs derived from early recurrent PDAC patients. In parallel, glutamine, glutamate, and aspartate were also starkly elevated in the early recurrence group. These metabolites are in line with the identified pathways from Figure 3b and Table 2. 

**Figure 4:**
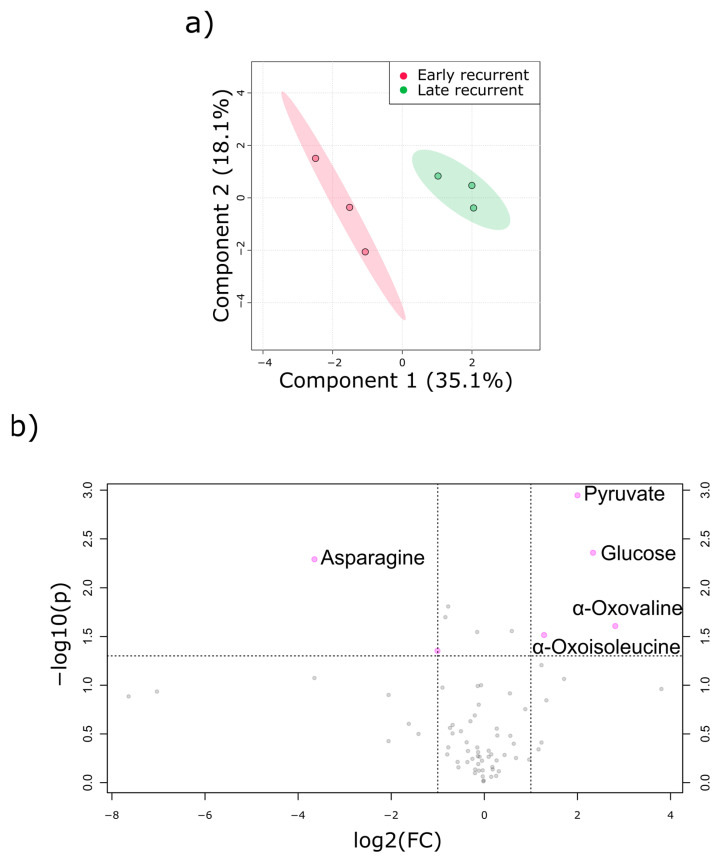
Exometabolome analysis of PDAC derived PDOs. (**a**) PLS-DA revealed discrimination between metabolites excreted from early recurrent PDOs (red) and late recurrent PDOs (green). (**b**) Volcano plot analysis of extracellular metabolites. Early recurrent to late recurrent fold changes are displayed as log2 values. Only top five metabolites are labeled. Each data point represents at least three technical replicates of the different PDOs.

In addition to the PDOs themselves, the PDO-conditioned culture medium was subjected to metabolic profiling. PLS-DA revealed again a discrimination between early and late recurrence (Figure 4a). The hypothesis of anaplerotic TCA-cycle activity in early recurrent PDAC could be substantiated by volcano plot analysis (Figure 4b) with the following observations: First, culture medium of PDOs derived from late recurrent PDAC patients had less glucose, which indicates that they relied more on glucose as an energy source. Second, asparagine was reduced in the medium of early recurrent PDOs, suggesting a higher import. Third, elevated secretion of α-keto carboxylic acid, including branched-chain ketoacids (BCKAs), in the early recurrence group indicates a higher activity of transaminases. BCATs (BCKA amino transferases) use branched chain amino acids to produce glutamate and BCKAs and ALT (alanine amino transferase) uses alanine to form glutamate and pyruvate. Therefore, these results are also in line with the highly increased intracellular glutamate concentration in early recurrent PDOs.

## 4. Discussion

In this study, we successfully generated and validated patient-derived organoid cultures from PDAC patients, who went on to developed recurrence after surgical resection either within six months (early recurrence) or later than six months (late recurrence). We demonstrated tissue identity of these PDO cultures by both mRNA analysis and immunohistochemistry analysis of typical lineage markers. ddPCR unraveled high MAFs of *KRAS* in all PDOs, further validating our cultures as cancer organoids. To the best of our knowledge, this is the first study comparing metabolites in PDOs from these two distinct, clinically significant PDAC subgroups. Of note, organoids of all patients were generated from resected primary biopsies at the time of operation when further disease course was still obscure. We could uncover higher levels of anaplerotic amino acids and TCA-cycle intermediates in the early recurrence group. In addition, the analysis of exometabolites unveiled elevated levels of α-keto acids and glucose together with decreased levels of asparagine in medium conditioned by early recurrent PDOs. Dufour and colleagues showed that PDAC bears in general low expression of asparagine synthetase and therefore is in high need for extracellular asparagine [53]. They suggested that an asparagine-low diet could have beneficial effects for PDAC patients. As we could show diminished levels of asparagine, we speculate that such an asparagine starvation could also delay PDAC recurrence after surgical resection. 

Higher extracellular levels of glucose in early recurrent PDAC indicate a decreased dependency on glycolysis in comparison to late recurrent PDAC PDOs. Viale et al. have already proposed that a PDAC sub-population could be responsible for PDAC relapse and elegantly showed an increased reliance on the TCA-cycle within this population [54]. As a consequence, they proposed that targeting the TCA-cycle might be a potential treatment option for PDAC therapy. Therefore, we think that in agreement with our results (higher extracellular glucose and higher intracellular TCA-cycle intermediates) these metabolic alterations might not only serve as a clinically applicable biomarker of early versus late disease recurrence but also as a therapeutic target to delay recurrence after surgical therapy. Of note, we here present a pilot study with low sample numbers and larger prospective trials are needed to confirm our findings in a larger patient population.

The laboratory of Joshua Rabinowitz compared metabolite concentrations in pancreatic cancer tissue with adjacent benign tissue and uncovered relatively higher levels of glutamic acid in pancreatic malignant tissue [55]. However, the amino acids which were relatively lower were nitrogen donors. Therefore, they speculated that increased deamination led to the opposing abundance of specific amino acids. In another study, they showed that in almost all tissues the TCA-cycle is mainly fueled by lactate, with the exception of the pancreas, which uses primarily glutamine/glutamate for this purpose [56]. These results are again in line with our observation of increased consumption of nitrogen donors and increased intracellular glutamate levels in early PDAC PDOs, which can be seen as more malignant tissue in comparison to PDOs derived from late recurrent PDAC patients. 

In humans, deamination reactions are normally catalyzed by transaminases, in which an amine group of an α-amino acid is transferred, resulting in glutamic acid and an α-keto carboxylic acid. Not only have we detected elevated levels of glutamic acid within the early recurrent PDO-cultures, but also have we detected several α-keto carboxylic acids in those supernatants. Therefore, it is reasonable that higher transaminase activities also go along with an earlier relapse of PDAC. Confirmatory, Li et al. have proven high levels of mitochondrial branched-chain amino acid aminotransferase (BCAT2) in human PDAC. Subsequently, they showed in a Kras-driven PDAC mouse model that pancreas weight is significantly reduced in *bcat2*^−/−^ PDAC mice [57]. Whereas BCAT2 is elevated in PDAC, enzymes metabolizing branched chained keto acids (BCKAs), i.e., BCKA-dehydrogenases are simultaneously decreased [58]. This further explains the elevated levels of BCKAs found in PDO-conditioned medium in the early recurrence group.

Taken together, common features of malignant pancreas cancer, i.e., high transaminase and TCA-cycle activity, are even more distinctive in cancers from patients who suffer from early recurrence after resection. Therefore, targeting these pathways might prolong survival of PDAC patients after surgery in the future.

## 5. Conclusions

In this study, we generated PDOs from early and late recurrent PDAC patients and uncovered metabolic alterations, which suggest an increased TCA-cycle activity and increased transamination in early recurrent PDOs. These are common features in malignant transformations of pancreatic tissue and their higher manifestation in early recurrent PDAC PDOs present a possible therapeutic vulnerability to fight pancreatic cancer relapse in the future.

## Figures and Tables

**Table 1:** Patient and clinical data of PDO lines used in this study. The table summarizes early recurrent (ER) and late recurrent (LR) patients data including sex and age, as well as clinical data including tumor grading, KRAS mutation and state of tumor recurrence. Patient ER-1 received three cycles FOLFIRINOX prior to resection, while all other patients did not receive any neoadjuvant chemotherapy.

Organoid Line	Sex	Age/Years	TNM	CRM	Tumor Size/cm	Tumor Location	PDAC Grading	KRAS Mutation	Time to, Location of Recurrence/days	Survival (Last Follow Up) after Resection/days
ER-1	Male	32	pT3, pN1 (3/14). L0. V0. Pn1	R1	6.5	Pancreas head	G2	G12R	101, Liver	(1172)
ER-2	Male	67	pT3, pN1 (7/27). L1. V0. Pn1	R1	4.5	Pancreas head	G2	G12D	69, Liver	(1106)
ER-3	Female	66	pT3, pN2 (5/37). L1. V1. Pn1	Narrow	5.0	Pancreas body	G2	G12D	42, Liver	94
LR-1	Male	77	pT3, pN2 (6/16). L1. V0. Pn1	Narrow	5.0	Pancreas head	G3	G12R	538, Lung	886
LR-2	Male	72	pT2, pN2 (4/22). L1. V0. Pn1	Narrow	3.8	Pancreas head	G3	G12D	431	(431)
LR-3	Male	58	pT2, pN1 (1/19). L1. V0. Pn0	R1	3.2	Pancreas head	G2	G12D	492, Liver local	(543)

**Table 2:** Pathway entities with quality criteria and statistics from analysis shown in Figure 3b.

Pathway Abbreviation	Pathway	Pathway Impact	−log(p)
(a)	Alanine, aspartate and glutamate metabolism	0.6234	9.4709
(b)	beta-Alanine metabolism	0.39925	7.5625
(c)	Pyruvate metabolism	0.32192	7.0445
(d)	Histidine metabolism	0.22131	5.7457
(e)	TCA cycle	0.24338	4.7773
(f)	Glutamine and glutamate metabolism	0.5	4.8607
(g)	Starch and sucrose metabolism	0.55921	4.4369

## Supplementary Materials

The following are available online at https://www.mdpi.com/2072-6694/12/6/1440/s1, Figure S1: Mutation allele frequencies of KRAS, Table S1: Primer sequences used in this study, Table S2: Antibodies used in this study, Table S3: Results of endometabolic profiling, Table S4: Results of exometabolic profiling.
