# Supplementary material for: Metabolic Profiling of Early and Late Recurrent Pancreatic Ductal Adenocarcinoma Using Patient-Derived Organoid Cultures
Source: Cancers (Basel). 2020 Jun 1;12(6):1440. doi: 10.3390/cancers12061440 (PMC7352957; doi:10.3390/cancers12061440)
Supplement: Supplementary file 1 [file cancers-12-01440-s001.zip › cancers-815420 - supple-final/cancers-815420 - supplementary - final.pdf]

cancers

Supplementary Materials

Metabolic profiling of early and late recurrent pancreatic ductal adenocarcinoma using patient-derived organoid cultures

Lukas M. Braun, Simon Lagies, Rhena F.U. Klar, Saskia Hussung, Ralph M. Fritsch, Bernd Kammerer and Uwe A. Wittel

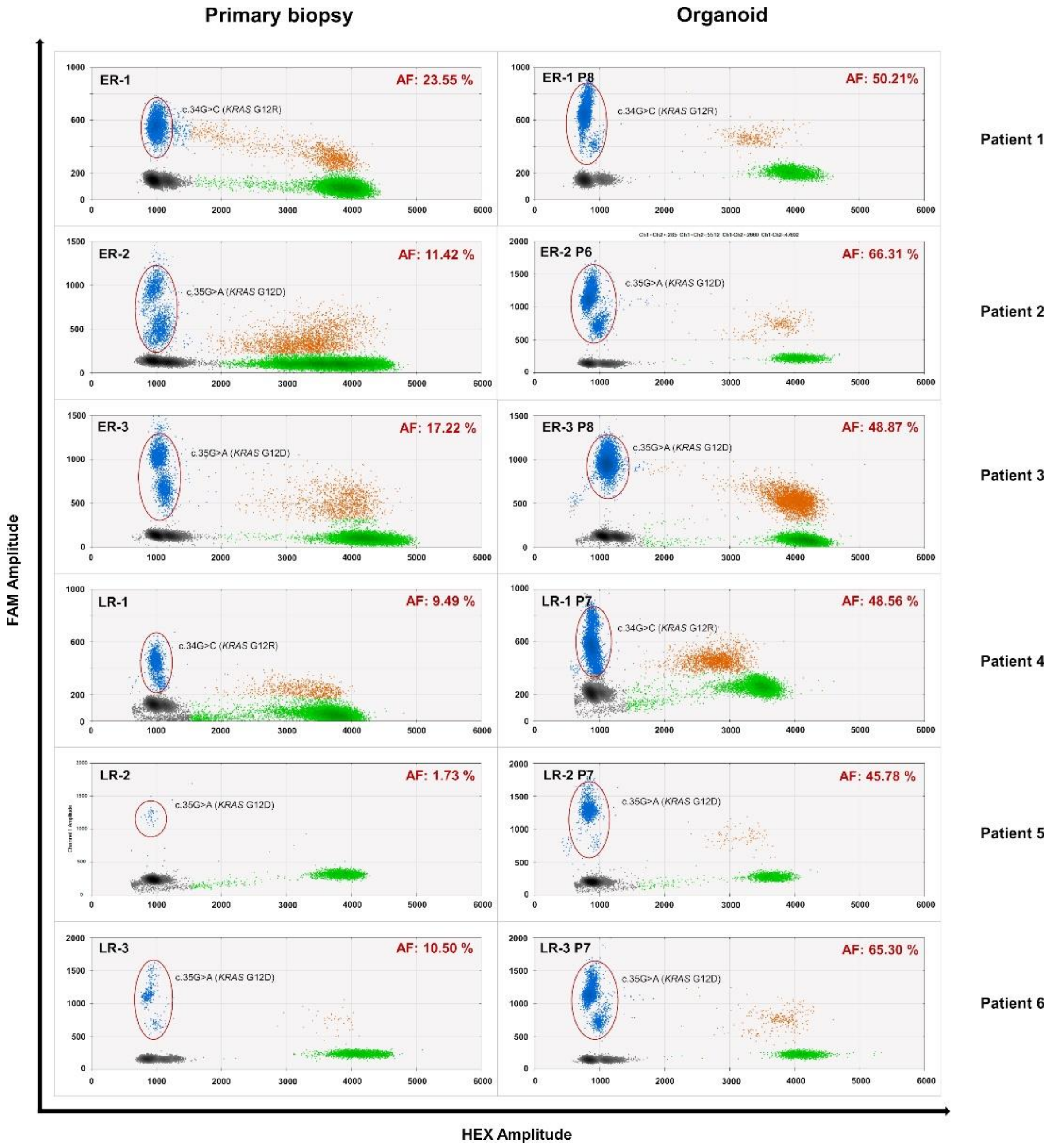

Figure S1. Mutation allele frequencies of KRAS. All PDOs are very pure tumor population and highly enriched in comparison to the primary biopsies as proven by ddPCR. ER: early recurrent, LR: late recurrent, Blue: droplets positive for mutant KRAS, Green: WT KRAS droplets, Orange: double positive droplets, Gray: empty droplets. Each plot represents the mean of four technical replicates.

Table S1. Primer sequences used for qPCR analyses in this study. All primers were purchased from Sigma.

Gene	Sequence
ACTB (NM_001101.5)	
Forward	GCCCTGAGGCACTCTTCCA
Reverse	TTGCGGATGTCCACGTCA
ACTA2 (NM_001141945.2)	
Forward	TTCCAGCCATCCTTCATCGG
Reverse	TCTCCTTCTGCATTCGGTCG
KRT19 (NM_002276.5)	
Forward	CAGCCGGACTGAAGAATTGAAC
Reverse	CAGGTCAGTAACCTCGGACC
SOX9 (NM_000346.4)	
Forward	TCTGGAGACTTCTGAACGAGAG
Reverse	CTTGAAGATGGCGTTGGGGG
VIM (NM_003380.5)	
Forward	CTCCCTGAACCTGAGGGAAAC
Reverse	TTGCGCTCCTGAAAAACTGC

Table S2. Antibodies used for IHC analyses in this study.

Antibody	Species	Clone	Isotype	Supplier
Anti-αSMA	Mouse	1A4	IgG2a	Abco
Anti-CK19	Mouse	A53-B/A2.26 (Ks19.1)	IgG2a	Thermo Fisher Scientific
Anti-PDX1	Rabbit	EPR3358(2)	IgG	Abco
Anti-Vimentin	Mouse	V9	IgG1	DAKO
EnVision+/HRP. Mouse	NA	NA	NA	DAKO
EnVision+/HRP. Rabbit	NA	NA	NA	DAKO

Table S3: Results of endometabolic profiling, Table S4: Results of exometabolic profiling. Detailed information can be found at supplementary excel.

© 2020 by the authors. Submitted for possible open access publication under the terms and conditions of the Creative Commons Attribution (CC BY) license (http://creativecommons.org/licenses/by/4.0/).
